# A Sensitive Assay for Unbound Docetaxel Using Ultrafiltration plus HPLC-MS and Its Application to a Clinical Study

**DOI:** 10.3390/pharmaceutics16050602

**Published:** 2024-04-29

**Authors:** David Wang, Natalie Hughes-Medlicott, Lilian Klingler, Yi Wang, Noelyn Hung, Stephen Duffull, Tak Hung, Paul Glue, Albert Qin, Rudolf Kwan, Wing-Kai Chan, Christopher Jackson

**Affiliations:** 1 Department of Anaesthesia, Waikato Hospital, Hamilton 3204, New Zealand; 2 School of Pharmacy, University of Otago, Dunedin 9016, New Zealand; 3 Zenith Technology Limited, Dunedin 9016, New Zealand; 4 Department of Pathology, University of Otago, Dunedin 9016, New Zealand; 5 Certara, Radnor, PA 19087, USA; 6 Department of Psychological Medicine, University of Otago, Dunedin 9016, New Zealand; 7 PharmaEssentia Corporation, Taipei 115, Taiwan; 8 Athenex Limited, Buffalo, NY 14203, USA; 9 Department of Medicine, University of Otago, Dunedin 9016, New Zealand

**Keywords:** unbound assay, docetaxel, clinical trial

## Abstract

Introduction: Docetaxel, a taxane used in the treatment of solid tumours, exerts pharmacological activity when in its unbound form. We report a sensitive assay to quantify unbound docetaxel after oral administration of docetaxel plus encequidar (oDox+E). Unbound drug quantification is important due to its direct correlation with drug-related toxicity and therapeutic efficacy. We improve on the sensitivity of current assay methods and demonstrate the utility of the assay on a novel formulation of oral docetaxel. Methods: Ultrafiltration followed by high-performance liquid chromatography and tandem mass spectrometry (HPLC-MS/MS) was utilized. Long-term stability, precision, accuracy, and recovery experiments were conducted to validate the assay. Additionally, patient samples from a Phase I dose-escalation pharmacokinetic study were analyzed using the developed assay. Results: The assay method exhibited long-term stability with an observed change between 0.8 and 6.9% after 131 days of storage at −60 °C. Precision and accuracy quality controls met the FDA acceptance criteria. An average recovery of 88% was obtained. Patient sample analysis demonstrated successful implementation of the assay. Conclusion: A validated sensitive assay was developed with an LLOQ of 0.084 ng/mL using 485 µL of human plasma. The sensitivity of the assay allowed quantification of unbound docetaxel concentrations in an early-phase oDox+E clinical study to compare it against IV docetaxel using pharmacokinetic modelling. Successful development of oDox+E represents an opportunity to replace the current IV docetaxel regimen with an oral regimen with lower cost, decreased side effects, and improve patient quality of life and experience.

## 1. Introduction

Docetaxel is a taxane used in the treatment of a variety of solid tumours [1], and it is highly protein-bound to albumin and alpha 1-acid glycoprotein (AAG) in plasma [2]. Most docetaxel pharmacokinetic models are focussed on the total plasma concentration [3]. However, the activity of a drug directly relates to the free or unbound concentration of drug in plasma. Unbound drug in plasma is available to partition to the site of action of the molecular target and subsequently bind to the target, whereas bound drug is unable to reach the molecular target and exert an effect [4]. The unbound exposure of docetaxel has been shown to correlate better with drug-related haematological toxicity than to the total exposure of docetaxel [5]. Furthermore, the unbound fraction of taxanes has been shown to correlate more closely to the pharmacological effects than total plasma concentrations [6]. The unbound concentration of drug can be isolated using a variety of methods including equilibrium dialysis, ultrafiltration, ultracentrifugation, micro-partitioning, and biosensor-based analysis. The method used to isolate the unbound portion of drug is then followed by an assay to quantify the drug.

Due to poor bioavailability, docetaxel is administered intravenously (IV) [7]. Furthermore, due to poor aqueous solubility, polysorbate 80 (Tween 80) and ethanol are required in the intravenous formulation. Tween 80 can lead to hypersensitivity reactions necessitating pre-medication and fluid retention [8]. Many projects have investigated the feasibility of oral docetaxel due to the potential benefits to patients and the cost of healthcare delivery. An oral formulation avoids the need for IV access and time spent at a day stay unit [9]. It also avoids the co-administration of Tween 80, which can cause hypersensitivity reactions [8], and may reduce the financial burden required to deliver chemotherapy [10].

Encequidar is a novel, well-tolerated intestine-specific P-glycoprotein inhibitor (with minimal systemic uptake) which increases the absorption of orally administered docetaxel [11]. oDox+E is a novel form of oral docetaxel that consists of oral docetaxel administered one hour after 15 mg of oral encequidar [12]. The total plasma concentration after oral oDox+E consists of protein-bound docetaxel and unbound docetaxel. In contrast, the total plasma concentration of IV docetaxel consists of protein-bound docetaxel, formulation-associated docetaxel, and unbound docetaxel [13]. Therefore, the quantification of unbound docetaxel, which exerts the pharmacological effect, is preferable.

Previous assays developed to quantify unbound docetaxel utilised equilibrium dialysis or ultrafiltration to separate the unbound docetaxel followed by HPLC-MS/MS [14,15,16]. These methods report a lower limit of quantification (LLOQ) of 0.1 ng/mL to 0.4 ng/mL [14,16,17]. Given the small starting dose of oDox+E (75 mg/m^2^), we aimed to develop a more sensitive assay to quantify smaller concentrations of unbound docetaxel.

The aim of this research was to develop a sensitive assay using ultrafiltration followed by high-performance liquid chromatography and tandem mass spectrometry (HPLC-MS/MS) to quantify unbound docetaxel after administration of oral docetaxel plus encequidar (oDox+E).

## 2. Materials and Methods

### 2.1. Chemicals and Reagents

Docetaxel (United States Pharmacopeia reference standard) and paclitaxel (internal standard, IS) of analytical grade of known purity were purchased from Polymed Therapeutics and Toronto Research Chemicals, respectively. Methanol (≥99.9% grade), ethanol (≥99.9% grade), acetonitrile (≥99.9% grade), tert-butyl methyl ether (≥99.9% grade), 2-propanol (≥99.9% grade), and formic acid (Suprapure, 98–100%) were purchased from Merck (Darmstadt, Germany). Purified water B.P. grade was purchased from Biomed (Auckland, New Zealand). The named solutions/solvents and their component reagents are shown in Table 1.

### 2.2. Equipment

The HPLC consisted of a Prominence CBM-20A controller (Shimadzu, Kyoto, Japan) with a Prominence nexera X_2_ SIL-30AC auto-sampler (Shimadzu, Japan), two Prominence HPLC pumps (Shimadzu, Japan), a Luna 5 um C18 100 Å column (Phenomenex, Auckland, New Zealand), a Prominence CTO-20A column oven (Shimadzu, Japan), and a Prominence DGU-20A5R degasser (Shimadzu, Japan). The detector was a QTRAP 6500 triple quadrupole LC-MS/MS system from AB SCIEX controlled by Analyst software version 1.6.2 (Woodlands Central Industrial Estate, Singapore). Nitrogen was used as nebulisation, curtain, and collision gas. Evaporation under nitrogen was conducted in a plate drier. Amicon Ultra 0.5 mL filters (Ultracel—10K) from Merck (Darmstadt, Germany) were used for ultracentrifugation. Eppendorf microcentrifuge 5415R was used as the thermostatically controlled centrifugation from Eppendorf (Hamburg, Germany).

### 2.3. Sample Preparation and HPLC-MS/MS Analysis

#### 2.3.1. Patient Sample Preparation

Patient samples were obtained from a phase one trial reported elsewhere [18], ethical approval was obtained from the New Zealand Health and Disability Ethics Committee (HDEC),. reference number 15/STH/182. A quantity of 485 µL of sampled plasma was pipetted into an Amicon Ultra 0.5 mL filter (Utracel—10K). This was left to incubate at 37 °C for one hour, followed by centrifugation at 6000× *g* for one hour at 37 °C. Then, 300 µL of the resulting filtrate was pipetted into a 96-well extraction plate, and 50 µL of internal standard (IS) at 360 ng/mL was added to the filtrate (resulting in an IS concentration of 51.43 ng/mL). The quality control and standard curve samples were added at this step to individual wells in the extraction plate. The wells were then vortexed for 2 min at 2000 rpm. Next, 600 µL of extraction solvent was added to the wells. The entire plate was then centrifuged at 4000 rpm for 15 min at 4 °C. A quantity of 480 µL of the supernatant after the centrifugation was aspirated and transferred to the injection plate. The supernatant was dried under a stream of nitrogen and then reconstituted with 150 µL of mobile phase. The injection plate was then vortexed for 10 min at 2000 rpm and centrifuged at 4000 rpm for 10 min at 4 °C. The injection plate was then ready for HPLC-MS. The injection volume was 20 µL.

#### 2.3.2. Quality Control, Standard Curve, and Test Solution Preparation

Quality control and standard curve samples underwent the same preparation as the patient samples above and were added to the extraction plate. Test solutions (as described in Table 1) were added to the injection plate after reconstitution of the dried supernatant as indicated above.

#### 2.3.3. High-Performance Liquid Chromatography and Mass Spectrometry

Gradient elution was performed on a Luna 5 μm C18_(2)_ (150 mm × 2.00 mm id, 100 Å) column with mobile phases A and B (described above) at a flow rate of 0.5 mL/min. Turbo-spray ionisation in positive mode with multiple reaction monitoring was utilised. The mass spectrometry parameters and conditions that were optimised for this experiment are shown in Table 2. The typical retention time was 1.82 and 1.84 min for docetaxel and paclitaxel, respectively. The minimum intensity was set at 2.8 × 10^5^ and 2.6 × 10^5^ counts per second (CPS) for docetaxel and paclitaxel, respectively.

### 2.4. Method Validation

Long-term stability for docetaxel after 131 days of storage at −60 °C was quantified for three concentration levels each with six repeats, an observed change of less than 15% was deemed acceptable. Precision and accuracy of the assay were determined within and between days over three separate days at the five quality control concentration levels; Equations (1) and (2) show the calculation of precision and accuracy. As per FDA guidelines [19], the acceptance criteria for precision and accuracy were values not exceeding 20% for the lower limit of quantification (LLOQ) and 15% for all other quality control concentrations. Recovery and extraction experiments were carried out with three repeats for each experiment.

Equation (1) Precision formula:

(1) $$CV(\%)=\frac{s}{\bar{x}}\times 100$$

where CV is the coefficient of variation (representing precision), s is the standard deviation of determined concentrations for a given QC concentration, and $\bar{x}$ is the mean value of determined concentrations for a given QC concentration.

Equation (2) Accuracy formula:

(2) $$Accuracy(\%)=\frac{\sum_{i=1}^{N}\frac{\left|D_{i}-S\right|}{S}\times 100}{N}$$

where $D_{i}$ is the determined concentration, S is the nominal concentration, and N is the number of repeats for a given QC level.

#### 2.4.1. Quantitative analysis of patient samples

Nine patients with metastatic prostate cancer were recruited in a Phase I dose-escalation pharmacokinetic study for oDox+E [12]. Each patient had 24 and 23 plasma samples taken after administration of standard of care IV docetaxel and oDox+E, respectively. Seven to eight samples for each route of administration for each patient were chosen using optimal design [20] to undergo unbound concentration analysis.

#### 2.4.2. Assay Compliance during Patient Sample Analysis

Assay compliance during analysis of patient samples was quantified during each run through the inclusion of (1) two repeats of the standard curve at the beginning and the end of each assay to ensure linearity, (2) a triplicate of test solutions to evaluate the consistency of the retention time of docetaxel and the IS, and (3) two sets of five quality control samples interspersed between analysed samples to ensure accurate and precise measurements.

## 3. Results

### 3.1. Long-Term Stability

The average observed change in docetaxel after long-term storage was between 0.8 and 6.9%, as shown in Table 3. These met the criteria of an observed change of less than 15%.

### 3.2. Precision and Accuracy

Five quality control levels were repeated six times each day for three days. In total, 18 samples at each quality control level (QC) were available for analysis. The overall precision and accuracy fulfilled the FDA acceptance criteria of not exceeding a value of 20% for LLOQ and 15% for all other QCs, as shown in Table 4.

### 3.3. Recovery

The average recovery of docetaxel from the test solution after ultracentrifugation was 88% and is shown in Table 5. This indicates that non-specific binding was 12% for a spiked docetaxel concentration of 82 ng/mL with ethanol as the media. This concentration was chosen based on a conservative estimate of the likely peak concentrations of oDox+E given at a dose of 75 mg/m^2^.

### 3.4. Assay Compliance during Patient Sample Analysis

The test solution was measured three times in every run, and the consistency of the retention time for docetaxel, paclitaxel, and the peak area ratio (PAR) were evaluated. The CVs for the retention times were all below the acceptance criteria of 5%, ranging from 0.06% to 0.31% for docetaxel and 0.03% to 0.33% for the internal standard. The CVs for the PAR of docetaxel were also all well below the acceptance criteria of 10%, ranging from 0.8% to 2.8%.

In total, 90 QC samples were included in the 9 runs. Eighty-five out of the ninety samples met the acceptance criteria of less than ±15% variation from the nominal QC level, except for LLOQ where a ±20% variation was acceptable. No run had more than 1 QC sample that failed this criterion, demonstrating the accuracy of the assay. The margin of error for those that did not meet the acceptance criteria was between 0.2 and 3.3%.

In total, 198 standard curve samples were measured during patient sample analysis, and 197 of these samples met the acceptance criteria of actual concentrations within ±15% of the nominal concentration except for the 0.084 ng/mL level, where it should be within ±20%. Only one standard curve sample was outside of this range at a 16.7% deviation. All standard curves had a correlation coefficient value of >0.99. This confirmed the linearity of the standard curve model and the acceptable use of the standard curve to back-calculate the concentration of docetaxel in the samples of interest.

### 3.5. Quantitative Analysis of Patient Samples

**Figure 1:**
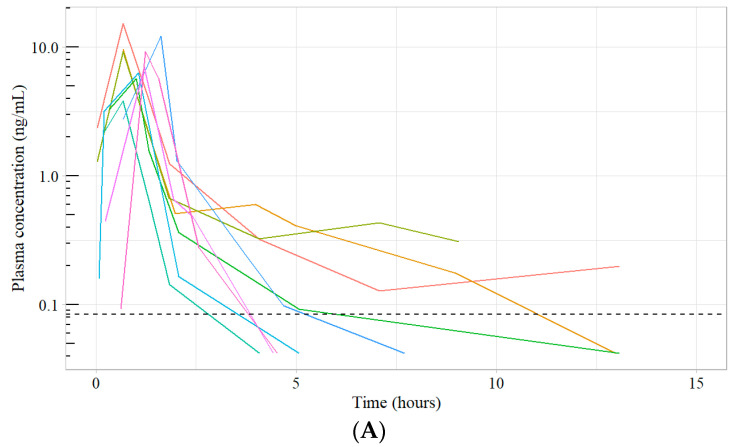

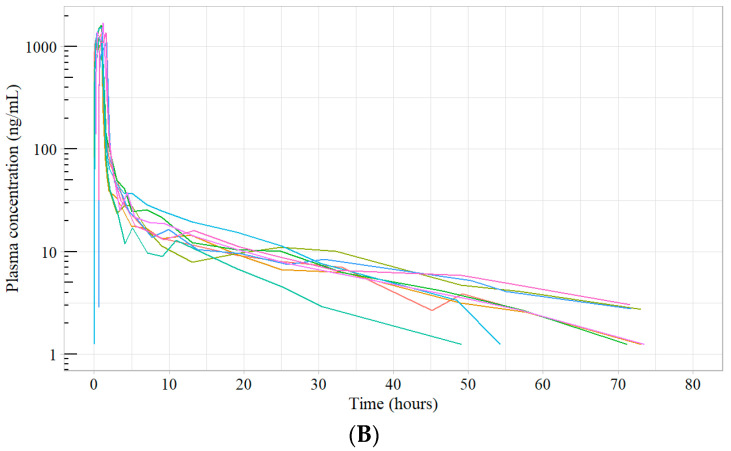
(**A**) Unbound docetaxel concentration after IV docetaxel administration (~75 mg/m^2^ over 1 h) in 9 patients (Dotted line represents 0.084 ng/mL). (**B**) Total docetaxel concentration after IV docetaxel administration (~75 mg/m^2^ over 1 h) in 9 patients. Coloured lines represent each patient.

**Figure 2:**
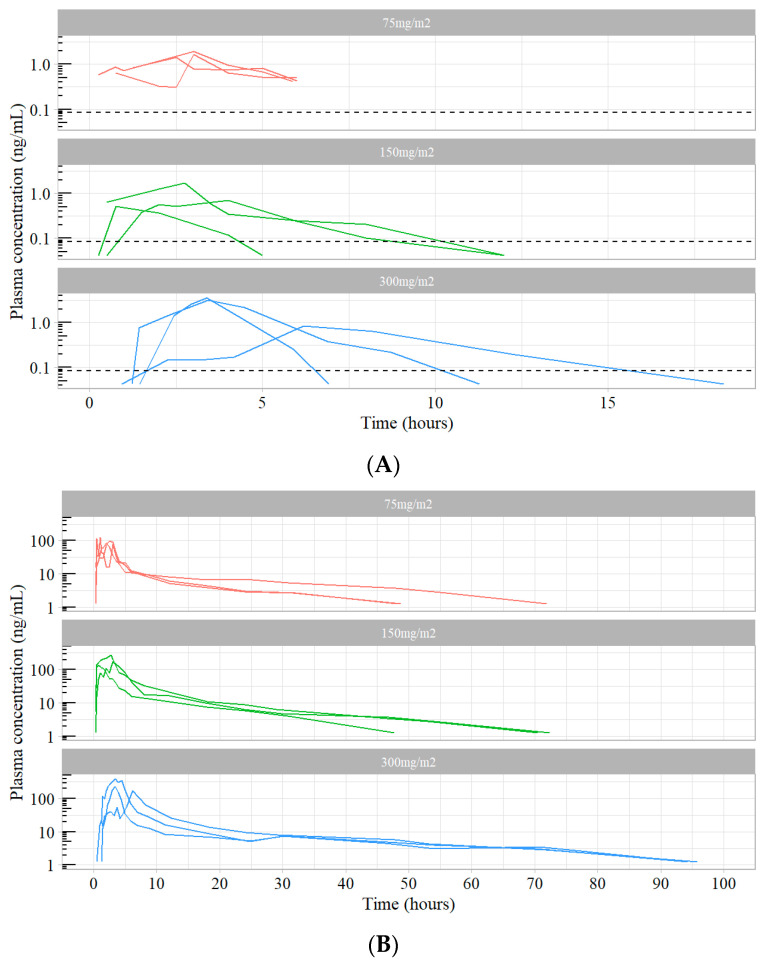
(**A**) Unbound docetaxel concentration after oDox+E administration by dose level (Dotted line represents 0.084 ng/mL). (**B**) Total docetaxel concentration after oDox+E administration by dose level.

Seven to eight samples after IV docetaxel administration and oDox+E administration were selected for unbound docetaxel analysis from each patient recruited in the Phase I trial [12]. The assay was implemented on these samples, and the quantified unbound docetaxel concentrations and the corresponding total docetaxel concentrations are shown in Figure 1 and Figure 2. The average fraction unbound across all measured samples were 1.3% and 0.7% after oDox+E and IV docetaxel administration, respectively.

## 4. Discussion

Validated methods to quantify unbound docetaxel have been reported previously; however, the lowest level of quantification was 0.1–0.4 ng/mL [16,17]. Here, we report the successful development of a slightly more sensitive assay to quantify unbound docetaxel with an LLOQ of 0.084 ng/mL in plasma samples from patients receiving oDox+E. The sensitive assay allows comparison of alternative formulations of docetaxel to the current standard of care IV docetaxel and aids in the development of these formulations which likely have a different fraction unbound compared to IV docetaxel, as demonstrated in the results.

The precision and accuracy of the developed assay were assessed to ensure reliable and accurate quantification of docetaxel. Five quality control levels were analysed six times on three occasions, resulting in a total of 18 samples at each quality control level. The precision, represented by the coefficient of variation (CV), and accuracy met the acceptance criteria of not exceeding 20% for the lower limit of quantification (LLOQ) and 15% for all other quality control levels. Recovery experiments were conducted to evaluate the efficiency of the ultracentrifugation process for separating docetaxel from the test solution. The average recovery of docetaxel was determined to be 88%, indicating that 12% of the docetaxel experienced non-specific binding. Assay compliance during patient sample analysis was carefully monitored to ensure the accuracy and reliability of the results. The retention times of docetaxel, paclitaxel (internal standard), and the peak area ratio (PAR) were evaluated to assess the consistency of the measurements. The CVs for the retention times were all below the acceptance criteria of 5%, indicating the stability and reproducibility of the chromatographic system. Similarly, the CVs for the PAR of docetaxel were well below the acceptance criteria of 10%.

The developed assay was successfully implemented for the quantitative analysis of patient samples from a Phase I dose-escalation pharmacokinetic study for oDox+E reported elsewhere. Unbound docetaxel concentrations were quantified and compared to the corresponding total docetaxel concentrations. The results showed that the average fraction of unbound docetaxel was 1.3% and 0.7% after oDox+E and IV docetaxel administration, respectively. A limitation to the assay was that it was still insufficiently sensitive enough to detect plasma concentrations of drug accurately at the lowest oDox+E dose of 75 mg/m^2^ around 7 h post administration. This was not unexpected, as the starting dose was purposefully chosen to be very low as the study was first-in-human, and subsequent dose levels were successfully quantified.

The oncology drug development paradigm is shifting with the introduction of Project Optimus by the FDA [21]. There is a renewed focus on the use of dose selection and optimisation at the earliest stage possible of drug development. The development of a more sensitive assay for unbound docetaxel allows more refined dose selection and optimisation of novel formulations (as total concentration comparisons do not take into account differences in fraction unbound). This assay was applied in retrospect to optimally selected samples to allow development of a docetaxel pharmacokinetic model which includes total, unbound, IV docetaxel and oDox+E data. The model developed allowed simulations to be performed to make a GO/NO-GO decision and propose a dosing regimen [18]. Overall, the development of this assay facilitated the oDox+E development process. In future, it may further facilitate the development of novel docetaxel formulations and allow more precise therapeutic drug monitoring to individualised dosing regimens.

These findings demonstrate the robustness, accuracy, and applicability of the developed assay for the analysis of docetaxel in plasma samples. It showed the difference in unbound concentration between oDox+E and IV docetaxel that could not be accounted for if total concentration alone was measured. The assay contributed to a better understanding of the pharmacokinetics of docetaxel. Ultimately, it aided in optimizing dosing regimen of oDox+E and will be applicable to future development of novel formulations and subsequent trials in patients with different oncology diagnoses.

## 5. Conclusions

Measurement of the unbound plasma docetaxel is important after the administration of different docetaxel formulations. A validated sensitive assay was developed with an LLOQ of 0.084 ng/mL using 485 µL of human plasma. The improved sensitivity allowed quantification of unbound docetaxel concentrations for oDox+E to facilitate development of a pharmacokinetic model used for dose regimen selection. A sensitive assay of unbound docetaxel facilitates dose selection and optimisation in early clinical drug development of new formulations of docetaxel.

## Figures and Tables

**Table 1:** Named solutions/solvents and their component reagents.

Solution/Solvent	Reagent(s)
Reconstitution solution	Acetonitrile:water (20:80, *v*/*v*) with 0.1% *v*/*v* formic acid
Extraction solvent	Tert-butyl methyl ether
Injection solvent	2-propanol
Mobile phase A	Acetonitrile:Water (5:95, *v*/*v*) with 0.1% *v*/*v* formic acid
Mobile phase B	Acetonitrile:Water (95:5, *v*/*v*) with 0.1% *v*/*v* formic acid
Standard curve	Spiked docetaxel (dissolved in ethanol) in human plasma to 0.084, 0.21, 0.524, 1.311, 3.277, 8.192, 20.48, 51.2, 128, 288, 320 ng/mL
Internal standard	Paclitaxel dissolved in ethanol–water (50:50, *v*/*v*) to 360 ng/mL
Test solution	Docetaxel at 82 ng/mL and paclitaxel at 96 ng/mL dissolved in ethanol.
Quality control samples	Docetaxel spiked in human plasma to 0.084 (LLOQ), 0.252 (Low QC), 8 (Med 1 QC), 160 (Med 2 QC) and 256 (High QC) ng/mL.

Abbreviations: *v*/*v*—Volume-to-volume ratio; LLOQ—Lower limit of quantification; Low QC—Low quality control concentration; Med 1 QC—First medium quality control concentration; Med 2 QC—Second medium quality control concentration; High QC—High quality control concentration.

**Table 2:** Mass spectrometry (A) source/gas parameters and (B) compound parameters.

(**A**)		
**Parameters**		
Curtain gas (psi)	30	
Collision gas (psi)	30	
Ion spray voltage (volts)	5500	
Temperature (°C)	300	
Ion source gas 1 (psi)	65	
Ion source gas 2 (psi)	30	
(**B**)		
**Parameters**	**Docetaxel**	**Paclitaxel**
Q1 mass (amu)	808.4	854.5
Q3 mass (amu)	527.2	285.0
Declustering potential (volts)	45	60
Entrance potential (volts)	4	5
Collision energy (volts)	13	14
Collision cell exit potential (volts)	14	8

**Table 3:** Long-term stability of docetaxel after 131 days of storage at −60 °C.

	Nominal Concentration (ng/mL)			
	7.5	32	140	2000
	Actual Concentration Day 131 (ng/mL)			
	N = 6	N = 6	N = 6	N = 6
Mean	8.0	32.5	144.8	1984.1
Standard deviation	0.41	2.9	9.4	100.5
Observed change (%)	6.9%	1.7%	3.4%	0.8%

**Table 4:** Overall precision and accuracy of quality control samples carried out over 3 days.

	Nominal Concentration (ng/mL)				
	LLOQ	Low QC	Med 1 QC	Med 2 QC	High QC
QC	0.084	0.252	8	160	256
	**Actual Concentration (ng/mL)**				
	**LLOQ**	**Low QC**	**Med 1 QC**	**Med 2 QC**	**High QC**
N	18	18	18	18	18
Mean	0.087	0.253	8.0	158.4	254.6
SD	0.011	0.024	0.64	12.1	13.8
CV	13.0%	9.4%	8.1%	7.6%	5.4%
Accuracy	10.3%	7.0%	6.9%	6.0%	4.4%

Abbreviations: QC—Quality control level; LLOQ—Lower limit of quantification; Low QC—Low quality control concentration; Med 1 QC—First medium quality control concentration; Med 2 QC—Second medium quality control concentration; High QC—High quality control concentration; N—number of samples; SD—standard deviation; CV—Prevision as represented by the coefficient of variation.

**Table 5:** Recovery of docetaxel after ultrafiltration at 82 ng/mL.

Concentration	82 ng/mL	82 ng/mL
Filtration	No	Yes
N	3	3
Mean PAR	2.9235	2.5736
SD PAR	0.1185	0.1290
Percentage (%)		88.03

Abbreviations: N—Number of samples; Mean PAR—Mean value of peak area ratio; SD PAR—Standard deviation of peak area ratios.

## Data Availability

The raw data supporting the conclusions of this article will be made available by the authors on request.
